# Report from Mongolia – How much do we know about the incidence of rare cases in less developed countries: a case series

**DOI:** 10.1186/1752-1947-2-358

**Published:** 2008-11-25

**Authors:** Martin W Dünser, Otgon Bataar, Albert H Rusher, Walter R Hasibeder, Ganbat Tsenddorj

**Affiliations:** 1 Department of Anesthesiology and Critical Care Medicine, Innsbruck Medical University, Anichstrasse 35, 6020 Innsbruck, Austria; 2 Department of Anesthesiology and Critical Care Medicine, Central State University Hospital, Ulaanbaatar, Mongolia; 3 Department of Surgery, Central State University Hospital, Ulaanbaatar, Mongolia; 4 Department of Anesthesiology and Critical Care Medicine, Krankenhaus der Barmherzigen Schwestern, 4910 Ried im Innkreis, Austria

## Abstract

**Introduction:**

Case reports are important instruments to describe rare disease conditions and give a rough estimation of their global incidence. Even though collected in international databases, most case reports are published by clinicians from industrialized nations and little is known about the incidence of rare cases in less developed countries, which are home to 75% of the world's population.

**Case presentation:**

We present seven patients who suffered from diseases which are either considered to be rare or have not yet been described before according to international databases, but occurred during a 5-month period in one intensive care unit of a less developed country. During the observation period, patients with a spontaneous infratentorial subdural hematoma (Asian, female, 41 years), general exanthema and acute renal failure after diesel ingestion (Asian, male, 30 years), transient cortical blindness complicating hepatic encephalopathy (Asian, female, 49 years), Fournier gangrene complicating acute necrotizing pancreatitis (Asian, male, 37 years), acute renal failure due to acetic acid intoxication (Asian, male, 42 years), haemolytic uremic syndrome following septic abortion (Asian, female, 45 years), and a metal needle as an unusual cause of chest pain (Asian, male, 41 years) were treated. According to the current literature, all seven disease conditions are considered either rare or have so far not yet been reported.

**Conclusion:**

The global incidence of rare cases may be underestimated by contemporary international databases. Diseases which are currently considered to be rare in industrialized nations may occur at a higher frequency in less developed countries. Reasons may not only be a geographically different burden of certain diseases, limited diagnostic and therapeutic facilities, but also a relevant publication bias.

## Introduction

Case reports are important instruments to describe rare diseases and give a rough estimation of their global incidence [1]. Although collected in international databases, most case reports are published by clinicians from industrialized nations. Little is known about the incidence of rare cases in developing countries which are home to 75% of the world's population [2]. In this case presentation, we describe seven patients suffering from diseases which are either considered to be rare or have not yet been described before but occurred during a 5-month period in one intensive care unit (ICU) in a less developed country.

## Case presentation

The eight-bed ICU is located in one of 12 university hospitals in the Mongolian capital of Ulaanbaatar and receives critically ill adult patients with surgical, medical and neurological pathologies. From 1 July until 13 November 2007, a total of 203 patients were treated.

An overview is presented of the clinical course of seven ICU patients with rare or so far unknown disease conditions each followed by a concise review of the current literature. Table 1 summarizes the demographic and clinical data of all the patients. Written informed consent to anonymously present their histories in this case series was obtained from all the patients or their next of kin.

**Table 1:** Characteristics of Patients

Patient	ICU Admission Diagnosis	Gender	Age	Chronic Disease	**SAPS II**[16]	**TISS 28**[17]	ICU LOS (days)	ICU Outcome
1	Acute Infratentorial SDH	F	41	none	10	13	3	survived

2	Intoxication with diesel	M	30	allergy to diesel	26	18	8	survived

3	Hepatic encephalopathy	F	49	liver cirrhosis	32	15	5	survived

4	Acute necrotizing pancreatitis	M	37	chronic pancreatitis, alcohol abuse	49	28	10	survived

5	Intoxication with Acetic Acid	M	42	alcohol abuse	56	30	1	died

6	HUS after Septic Abortion	F	45	alcohol abuse	38	20	7	survived

7	Foreign Body Extraction	M	40	none	7	18	3	survived

ICU, intensive care unit; SAPS, simplified acute physiology score; TISS, therapeutic intervention severity score; LOS, length of stay; SDH, subdural hematoma; HUS, hemolytic uremic syndrome.

### Patient 1 – Spontaneous infratentorial subdural hematoma

A 41-year-old Asian woman presented to the emergency department with acute severe headache starting after a 2-day history of diarrhea. Cranial computed tomography revealed an acute left-sided infratentorial subdural hematoma. A cerebral angiogram did not show any abnormalities. The patient denied recent trauma, intake of coagulation active drugs or herbs, or a known bleeding tendency. Plasma (prothrombin time, 10 s; activated partial thromboplastin time, 28 s) and cellular (platelet count, 165,000/microliter) coagulation parameters were normal. The patient was fully conscious but complained of nausea and vertigo. She was transferred to the ICU for neurologic monitoring and supportive therapy. Because of the non-compressive size of the hematoma, neurosurgical decompression was withheld. The patient was discharged from the ICU with significantly improved symptoms 3 days later.

Spontaneous subdural hematomas of the posterior fossa are very rare in adults without a history of trauma. Less than 20 cases have been reported in the literature [3]. Almost all were associated either with anticoagulation therapy or coagulatory defects.

### Patient 2 – General exanthema and acute renal failure due to diesel ingestion

**Figure 1:**
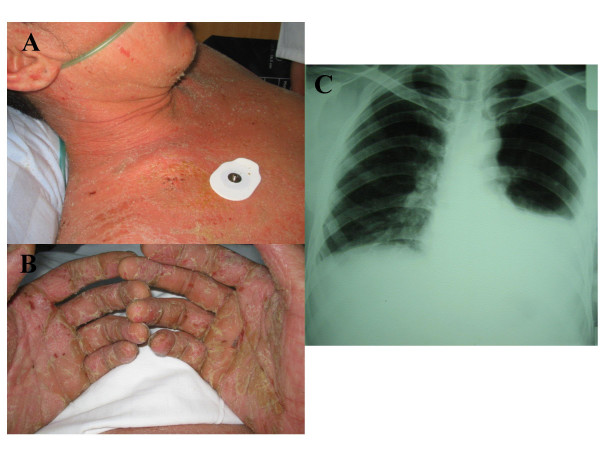
General exanthema (A, B) and left lower lobe pneumonia (C) after diesel ingestion.

During binge drinking, a 30-year-old Asian man with a known allergy to diesel (local skin reactions) ingested an unknown amount of diesel ('several sips') when siphoning fuel from a canister. Within hours he developed fever, chills, coughing and general exanthema (Figure 1A and 1B). On day two, hematuria developed and progressed into oliguria. After 7 days of cefazolin therapy because of pneumonia (Figure 1C) in a county hospital, the patient was admitted to the ICU with acute renal failure (creatinine, 740 μmol/liter). Except for mild respiratory insufficiency and metabolic acidosis (pH 7.29; standard bicarbonate, 13.5 mmol/liter; base deficit, -12 mmol/liter), he was stable. No history of cardiovascular instability could be evaluated. Liberal fluid resuscitation induced polyuria, decreased creatinine levels and evaded hemodialysis. Prednisolone was started at 80 mg and slowly tapered off after the general exanthema had improved.

Whereas localized dermatitis is known after diesel contact [4], no generalized exanthema following diesel ingestion has been reported. An allergic reaction seems to be the most probable cause in our patient. Acute renal failure has so far been observed in at least three patients after skin contact or diesel aspiration. Aliphatic hydrocarbon-induced hemolysis with hemoglobinuria, direct tubular toxicity, and allergic nephritis are possible pathogenetic mechanisms.

### Patient 3 – Transient cortical blindness complicating hepatic encephalopathy

A 49-year-old Asian woman with liver cirrhosis due to unspecified viral hepatitis was admitted to the ICU with coma (Glasgow Coma Scale, 9 pts; total bilirubin, 38 μmol/liter; blood sugar, 7 mmol/liter; arterial lactate, 2.5 mmol/liter; plasma albumin, 35 mg/dl). Loss of consciousness was preceded by diarrhea followed by gradual visual impairment. Cranial computed tomography, lumbar puncture and microbiological specimen were normal. In the electroencephalogram, triphasic waves and a delta rhythm were found. Two days after initiation of enteral lactulose (6 × 30 ml/day) and supportive treatment, the patient's conscious state improved. Ophthalmologic examinations at ICU admission and after the patient had regained full consciousness revealed no ocular pathology. One week later, the patient was discharged from the ICU with improved but still impaired vision.

Cortical blindness associated with hepatic encephalopathy was first described in 1979. Since then, only four case reports have been published. As in our case, visual disturbances preceded the loss of consciousness in all reports. The pathogenesis of hepatic blindness is unknown but may include hypotensive episodes and impaired blood brain-barrier function leading to cortical and subcortical edema [5].

### Patient 4 – Fournier gangrene complicating acute necrotizing pancreatitis

After binge drinking, a 37-year-old Asian man was admitted to the ICU with severe acute pancreatitis and multiple organ dysfunction (acute delirium; acute lung injury; total bilirubin 101 μmol/liter; creatinine 238 μmol/liter). His general condition improved with symptomatic ICU treatment. After a 10-day course of antibiotic prophylaxis (4 × 1 g cefotaxime/day), pancreas necroses remained sterile (fine needle puncture). After ICU discharge, the patient developed extensive necroses of the scrotum and perineum (Fournier gangrene) requiring repeated surgical necrosectomy.

So far, scrotal involvement has been reported as a complication of acute necrotizing pancreatitis in four patients [6]. According to the current literature, necrosis culminating in Fournier gangrene is a yet unknown complication of pancreatitis. Comparable to the patients experiencing necrosis of the scrotum, descending retroperitoneal necroses most likely resulted in Fournier gangrene in our patient.

### Patient 5 – Acute renal failure due to acetic acid intoxication

During binge drinking, a 42-year-old Asian man, a chronic alcoholic, involuntarily ingested ~100 ml of 80% acetic acid. After hospital admission, he developed intravascular hemolysis (hemoglobin, 57 g/liter; lactate dehydrogenase, 3752 IU/liter) and acute renal failure (creatinine, 1700 μmol/liter). When transferring the patient from the nephrological department to the ICU, he massively aspirated and died due to refractory pulmonary failure soon after ICU admission.

According to the experience of Mongolian physicians, acetic acid ingestion is a frequent intoxication requiring hospital admission. At least three cases of acetic acid-associated acute renal failure are observed in this hospital each year. In contrast, the current literature reports acute renal dysfunction to be a rare complication of acetic acid intoxication. So far, six case reports/series have been published. As in this patient, hemolysis with hemoglubinuria caused kidney injury in most patients [7], but direct toxic effects of acetic acid on renal tubules may also be involved [8].

### Patient 6 – Hemolytic uremic syndrome following septic abortion

A 45-year-old Asian woman suffered from septic abortion during gestational week 23. After curettage and initiation of antibiotic therapy (4 × 1 g ampicillin/day because of *E. coli *growing from an intrauterine swab), the patient was stable and free of organ dysfunctions. On postoperative day two, jaundice and oliguria developed, and she was transferred to the ICU. Despite fluid resuscitation, anuric renal failure (creatinine, 470 μmol/liter) developed. Repeated transfusions of red blood cells were required because of hemolytic anemia (hemoglobin, 61 g/liter; lactatedeyhdrogenase, 3027 IU/liter). Blood analysis revealed fragmentocytes and thrombopenia (platelets, 48,000/microliter). Intermittent hemodialysis was started. One week after ICU admission, the patient was discharged with rising erythrocyte and platelet counts. Because of persistent anuria, hemodialysis was continued for another 2 weeks. Subsequently, renal function gradually returned to normal.

Although extraintestinal causes of hemolytic uremic syndrome are known [9], only one case following septic abortion has been published [10]. Comparable to intestinal hemolytic uremic syndrome, the *E. coli *isolated from the uterine cavity in our patient not only caused abortion but most probably also hemolysis and acute renal failure. Although further laboratory specification of the pathogen was not possible, it is likely that the clinical condition was caused by Shiga toxin [9].

### Patient 7 – A metal needle as an unusual cause of chest pain

**Figure 2:**
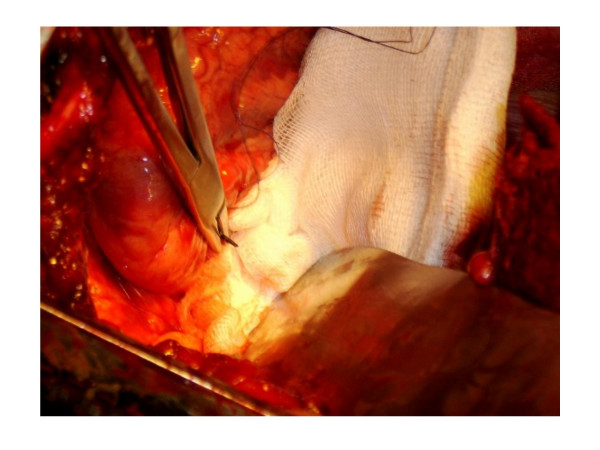
Intraoperative situs during the procedure to extract a metal needle lodged in the ventricular wall and causing subacute chest pain.

A 41-year-old Asian man presented to the emergency department with subacute recurrent chest pain. The electrocardiogram and biochemical laboratory parameters were normal. Chest fluoroscopy revealed a metal needle in the mediastinum. The patient could not remember having swallowed the needle. Since no part of the needle could be reached through endoscopy, the patient was scheduled for surgery. Choosing a left lateral thoracotomy, the needle could only partly be removed because scar tissue most probably had grown through the eye of the needle and prevented it from being extracted without causing damage to the ventricular wall (Figure 2). Adhesions between the posterior pericardium and the esophagus suggested that the needle had penetrated into the heart from the esophagus. The immediate postoperative course was complicated by respiratory problems but was uneventful afterwards. Three months after surgery, the patient still complained of intermittent mild chest pain.

Even though more than 100 cases of aortoesophageal fistulas have been reported, only a few cases of foreign body penetration into the heart are known [11].

## Discussion

According to the literature all disease conditions presented are either considered rare (Patients 1, 3, 5, 6, 7) or have not yet been reported (Patients 2, 4). Although no condition was observed more than once, the occurrence of seven such cases during a comparatively short time suggests an unusual accumulation, at least in the ICU evaluated. It is definitely possible that this is an unusual occurrence but it may also indicate that rare cases occur more frequently in less developed countries. This brings up the question whether the global incidence of certain disease conditions is underestimated by the current literature which is largely based on reports from highly developed countries. It is at least worthwhile to reflect on why certain diseases assumed to be rare could occur more often in less developed countries.

The higher disease burden in developing countries [12,13] makes the occurrence of unusual cases more likely. Moreover, certain diseases (for example, tropical diseases) are more frequent in the developing world. For example, the high incidence of chronic liver diseases in Mongolia [14] makes it probable that rare complications such as transient blindness associated with hepatic encephalopathy are observed more frequently. The widespread availability of potential toxins (for example, acetic acid) and the lack of adequate protective measures (for example, when handling fuel) result in a higher incidence of intoxications in less developed countries. Rare complications are further facilitated by variable diagnostic and therapeutic standards. It may be argued that earlier and more aggressive fluid resuscitation could have prevented acute renal failure in the two patients with acetic acid and diesel ingestion. Similarly, better imaging techniques and the possibility of performing interventional radiological procedures would have allowed earlier detection of the descending necroses and drainage in the pancreatitis patients.

Considering the low number of active scientists in developing countries [15], it is less likely that rare cases occurring in these regions will be published. This may be the reason why international databases suggest acute renal failure to be a rare complication of acetic acid poisoning while Mongolian physicians encounter this condition quite frequently. It may be hypothesized that further diseases remain either unknown or their global incidence underestimated simply because cases from less developed countries do not appear in international databases.

However, these points must not lead to the assumption that medical conditions which appear unclear in developing countries are rare cases that have not been observed in the medical literature. In contrast, it is much more likely that inadequate diagnostic facilities and limited educational standards preclude the diagnosis of well-known diseases. Given the possibility to perform appropriate diagnostic procedures and the clear disease presentation in our patients, it is unlikely that these cases are indeed "overlooked" common disease processes.

## Conclusion

The global incidence of rare cases may be underestimated by contemporary international databases. Diseases which are currently considered to be rare in industrialized nations may occur at a higher frequency in less developed countries. The reasons may not only be a geographically different burden of certain diseases, limited diagnostic and therapeutic facilities but also a relevant publication bias.

## Abbreviations

ICU: intensive care unit

## Consent

Written informed consent was obtained from the patients for publication of this case report and any accompanying images. A copy of the written consent is available for review by the Editor-in-Chief of this journal. In the case of patient 5 who died, consent for publication was sought from his next of kin.

## Competing interests

The authors declare that they have no competing interests.

## Authors' contributions

MWD made a substantial contribution to conception and design, interpreted the data and drafted the manuscript. OB gathered the data, interpreted the data and helped in drafting the manuscript. AHR gathered the data and helped in drafting the manuscript. WRH interpreted the data and helped in drafting the manuscript. GT made a substantial contribution to conception and design, interpreted the data and helped in drafting the manuscript. All authors read and approved the final manuscript.

## References

[B1] Vandenbroucke JP Case reports in an evidence-based world J R Soc Med 1999 92 159 163 10450190 10.1177/014107689909200401 PMC1297135

[B2] The Human Development Report 2007/2008 http://hdr.undp.org

[B3] Berhouma M Houissa S Jemel H Khaldi M Spontaneous chronic subdural hematoma of the posterior fossa J Neuroradiol 2007 34 213 215 17572494 10.1016/j.neurad.2007.05.004

[B4] Wahlberg JE 'Green diesel' – skin irritant properties of diesel oils compared to common solvents Contact Dermatitis 1995 33 359 360 10.1111/j.1600-0536.1995.tb02062.x 8565503

[B5] van Pesch V Hernalsteen D van Rijckevorsel K Duprez T Boschi A Ivanoiu A Sindic CJ Clinical, electrophysiological and brain imaging features during recurrent ictal cortical blindness associated with chronic liver failure Acta Neurol Belg 2006 106 215 218 17323839

[B6] Lin YL Lin MT Huang GT Chang YL Chang H Wang SM How SW Acute pancreatitis masquerading as testicular torsion Am J Emerg Med 1996 14 654 655 10.1016/S0735-6757(96)90081-9 8906763

[B7] Sangüesa Molina JR Macía Heras ML Acute oliguric kidney failure secondary to acetic acid poisoning An Med Interna 1999 16 9 461 462 10609359

[B8] Boseniuk S Rieger C Acute oral acetic acid poisoning – case report Anaesthesiol Reanim 1994 19 3 80 82 8086095

[B9] Blackall DP Marques MB Hemolytic uremic syndrome revisited: Shiga toxin, factor H, and fibrin generation Am J Clin Pathol 2004 121 S81 S88 15298153 10.1309/06W402EHNGVVB24C

[B10] Sens YA Miorin LA Silva HG Malheiros DM Filho DM Jabur P Acute renal failure due to hemolytic uremic syndrome in adult patients Ren Fail 1997 19 279 282 9101604 10.3109/08860229709026289

[B11] Medina HM Garcia MJ Velazquez O Sandoval N A 73-year old man with chest pain 4 days after a fish dinner Chest 2004 126 294 297 10.1378/chest.126.1.294 15249474

[B12] Mathers CD Loncar D Projections of global mortality and burden of disease from 2002 to 2030 PLoS Med 2006 3 442 10.1371/journal.pmed.0030442 PMC1664601 17132052

[B13] Ezzati M Lopez AD Rodgers A Hoorn S Vander Murray CJ Comparative Risk Assessment Collaborating Group Selected major risk factors and global and regional burden of disease Lancet 2002 360 1347 1360 10.1016/S0140-6736(02)11403-6 12423980

[B14] Oyunsuren T Kurbanov F Tanaka Y Elkady A Sanduijav R Khajidsuren O Dagvadorj B Mizokami M High frequency of hepatocellular carcinoma in Mongolia; association with mono-, or co-infection with hepatitis C, B, and delta viruses J Med Virol 2006 78 1688 1695 10.1002/jmv.20755 17063518

[B15] Langer A Díaz-Olavarrieta C Berdichevsky K Villar J Why is research from developing countries underrepresented in international health literature, and what can be done about it? Bull World Health Organ 2004 82 802 803 15643806 PMC2623037

[B16] Le Gall JR Lemeshow S Saulnier F A new Simplified Acute Physiologic Score (SAPS II) based on a European/North American multicenter study JAMA 1993 270 2957 2963 10.1001/jama.270.24.2957 8254858

[B17] Miranda DR de Rijk A Schaufeli W Simplified Therapeutic Intervention Scoring System: the TISS-28 items – results from a multicenter study Crit Care Med 1996 24 64 73 10.1097/00003246-199601000-00012 8565541

